# Study of the Synchronization and Transmission of Intracellular Signaling Oscillations in Cells Using Bispectral Analysis

**DOI:** 10.3390/biology13090685

**Published:** 2024-09-02

**Authors:** Maxim E. Astashev, Dmitriy A. Serov, Arina V. Tankanag, Inna V. Knyazeva, Artem A. Dorokhov, Alexander V. Simakin, Sergey V. Gudkov

**Affiliations:** 1 Prokhorov General Physics Institute of the Russian Academy of Sciences, Vavilove St. 38, 119991 Moscow, Russia; astashev@yandex.ru (M.E.A.); dmitriy_serov_91@mail.ru (D.A.S.); s_makariy@rambler.ru (S.V.G.); 2 Federal Research Center “Pushchino Scientific Center for Biological Research of the Russian Academy of Sciences”, Institute of Cell Biophysics of the Russian Academy of Sciences, 3 Institutskaya St., 142290 Pushchino, Russia; tav@pbcras.ru; 3 Federal Scientific Agroengineering Center VIM, 1st Institutsky Proezd 5, 109428 Moscow, Russia; knyazewa.inna@yandex.ru (I.V.K.); dorokhov-91@yandex.ru (A.A.D.); 4 Institute of Biology and Biomedicine, Lobachevsky State University of Nizhny Novgorod Institute, Gagarin av. 23, 603105 Nizhny Novgorod, Russia

**Keywords:** bispectral index, cell synchronization, calcium, nitric oxide, endothelial cells, heating, hyperglycemia, 42C40, 92C37

## Abstract

**Simple Summary:**

Fluctuations of physiological characteristics play an important role in a living organisms’ normal existence and adaptation to environmental conditions. An important feature of oscillations in living systems is their ability to form (synchronization) or break links (desynchronization) with each other. For example, synchronization/desynchronization in the brain’s electrical activity plays a role in memory and learning; synchronization changes cause epilepsy and other diseases. The task of quantifying the synchronization of signaling events in cells is currently unsolved. No universal, informative and accurate method has been found for assessing the synchronization of large cell groups. For the first time, we have tested the possibility of applying a mathematical method called bispectral analysis (applied earlier on the whole organism) to assess the coupling of signaling molecules and the level of fluctuations in cells. This method allows for a highly accurate estimation of the connection of oscillations between all pairs of cells in a studied population, the magnitude and frequency of these coupled oscillations, and which cells create and receive a “signal to form a connection of oscillations”. We have shown that stress (overheating and excessive glucose concentration) changes the synchronization of oscillations of signaling molecules in cells. The obtained data can be applied in medicine and human economic activity.

**Abstract:**

The oscillation synchronization analysis in biological systems will expand our knowledge about the response of living systems to changes in environmental conditions. This knowledge can be used in medicine (diagnosis, therapy, monitoring) and agriculture (increasing productivity, resistance to adverse effects). Currently, the search is underway for an informative, accurate and sensitive method for analyzing the synchronization of oscillatory processes in cell biology. It is especially pronounced in analyzing the concentration oscillations of intracellular signaling molecules in electrically nonexcitable cells. The bispectral analysis method could be applied to assess the characteristics of synchronized oscillations of intracellular mediators. We chose endothelial cells from mouse microvessels as model cells. Concentrations of well-studied calcium and nitric oxide (NO) were selected for study in control conditions and well-described stress: heating to 40 °C and hyperglycemia. The bispectral analysis allows us to accurately evaluate the proportion of synchronized cells, their synchronization degree, and the amplitude and frequency of synchronized calcium and NO oscillations. Heating to 40 °C increased cell synchronization for calcium but decreased for NO oscillations. Hyperglycemia abolished this effect. Heating to 40 °C changed the frequencies and increased the amplitudes of synchronized oscillations of calcium concentration and the NO synthesis rate. The first part of this paper describes the principles of the bispectral analysis method and equations and modifications of the method we propose. In the second part of this paper, specific examples of the application of bispectral analysis to assess the synchronization of living cells in vitro are presented. The discussion compares the capabilities of bispectral analysis with other analytical methods in this field.

## 1. Introduction

Oscillations in biological systems are defined as periodic processes of change in specific characteristics of a biological system. Examples at the cellular level include periodic alterations in protein and inorganic ion concentrations (e.g., circadian rhythms and brain biopotentials), the rate of cytoskeleton assembly and disassembly, and so forth [1,2]. The amplitude–frequency characteristics of oscillations in biological systems are typically subject to fine regulation by a number of mechanisms. Should a pair of oscillatory processes be regulated by a common mechanism, synchronization of oscillations may occur. Synchronization of oscillations can be defined as the appearance of phase coherence between periodic processes, expressed in the constancy of the phase overlap between the oscillations being analyzed. In a number of conditions, the phenomenon of synchronization can occur or, conversely, be lost. Desynchronization is the term used to describe the loss of synchronization. In the absence of synchronization, oscillations are referred to as desynchronized.

Periodic processes (or biorhythms) are oscillations that play a significant role in maintaining the homeostasis of living systems. Examples of periodic processes in humans and animals are numerous: rhythms of sleep and wakefulness, oscillations of hormone concentrations in the blood, expression of transcription factors, electrical activity of neurons and the heart, oscillations in the concentrations of intracellular mediators in cells, and other periodic processes [3,4,5,6,7,8,9,10,11,12,13]. Changes in human biorhythms are associated with a wide range of disorders and pathological conditions, in particular, insomnia, arrhythmia, and disorders of the blood supply to tissues [14,15,16,17,18,19,20,21]. A wide range of biorhythms have also been discovered in plants, e.g., femtosecond processes occurring during photosynthesis, minute processes regulating plant metabolism and tropisms, and daily and annual rhythms [22,23,24,25,26,27,28,29]. Understanding the mechanisms of regulation of plant biorhythms will make it possible to increase the productivity of agricultural crops and increase their resistance to unfavorable environmental factors.

In addition to the amplitude–frequency characteristics of rhythmic oscillations in one biological system, the synchronization of oscillatory processes in different tissues and organs is also important. In particular, it is the change in the synchronization of brain biopotentials that is considered a key stage in the processes of learning and memory [30]. Desynchronization or excessive synchronization of biorhythms can be the causes and/or markers of a number of human pathologies. For example, extreme changes in the synchronization of field potential oscillations between areas of the cerebral cortex can be the cause of a number of epilepsies or disorders, act as a marker of developmental defects, etc. [31,32,33,34,35,36]. Controlling the synchronization of EEG rhythms can be used to create optimal human–machine interfaces for use in the treatment of stress-induced conditions [37,38,39]. The degree of synchronization of fluctuations in the levels of biologically active substances in the blood serum (vitamins C and A, fasting blood sugar and malondialdehyde) can act as markers of the severity of type 2 diabetes mellitus [40].

There are many methods and approaches to analyzing the synchronization of oscillatory processes, each of which has its own advantages and disadvantages. The more common methods are the phase locking estimate, S estimator, global field synchronization, stochastic event synchrony, etc. [41,42,43,44,45,46,47,48,49]. However, the search for an “ideal” method is currently ongoing, which would work in a wide range of frequencies with high accuracy in amplitudes and frequencies, would have a minimal risk of false positive results, and would make it possible to determine the mechanism of synchronization. The method of bispectral analysis, which has been adapted and applied in order to synchronize additional parameters, may prove to be a promising approach for determining the synchronization of biorhythms [50]. The bispectral analysis could be successful at estimating oscillator coupling asymmetry. In other words, to determine the direction of transmission of oscillations (phases) between oscillators [51].

Rhythmic processes in cells play a significant role in regulating the functioning of animal and plant cells [52]. Oscillations of calcium and NO have been described for a wide range of cells: adipocytes, endothelial cells, neurons, cardiomyocytes and others [53,54,55,56,57]. However, work on the analysis of cell synchronization based on oscillations of intracellular mediators is, as a rule, limited to a qualitative description of synchronization without the use of spectral methods of mathematical processing [58,59]. Sometimes authors focus specifically on analyzing the waveforms of synchronized cells, but often synchronization is assessed qualitatively or semi-quantitatively and solely by expert judgment [60,61]. Works with quantitative mathematical analysis of the phase difference between the studied oscillatory processes are often limited to mathematical models and their analysis [62,63,64]. In works that combine mathematical modeling of intracellular mediator oscillations and analysis of experimental data, single recordings are usually analyzed and synchronization assessment is fundamentally impossible [65].

Contrastingly, we can highlight the work on calcium signaling in neuronal cultures or living brain slices. Some of the work performs excellent quantitative analysis of the synchronization of calcium oscillations of cells between different brain structures, different types of brain cells, or at different stages of neuronal development [66,67,68]. Unfortunately, in these cases, it is not possible to assess the direction of signal transmission from structure to structure. In classical electrophysiology of the brain, authors can also often determine the spatial distribution of synchronized areas, but cannot determine which of them is the “pacemaker” and which is the “receiver” [69]. Sometimes an approach is used to expertly analyze signal transmission from cell to cell in a complex culture of hippocampal neurons [70,71]. However, this approach is very labor-intensive, and its reproducibility may depend on the individual expert. We believe that the ability to determine the transmission of the synchronizing signal could be useful in classical in vivo neurophysiology using EEG.

Despite the fact that the processes of synchronization/desynchronization have been studied in detail at the organismal level, these phenomena have been scarcely studied at the cellular level. We believe that the key problem hampering research in this area is the lack of an adequate mathematical processing method with high accuracy and sensitivity. We show that the method of bispectral analysis can be successfully applied in assessing the synchronization of oscillation in the concentrations of signaling molecules in cells. Changes in the oscillations of intracellular calcium messengers Ca^2+^ and nitric oxide NO in electrically non-excitable cells are of interest due to their possible application in assessing the condition and treating pathologies of such body systems as the immune and cardiovascular systems [72,73,74]. In addition, when analyzing the oscillations of Ca^2+^ and other molecules in electrically non-excitable cells, expert assessment is significantly difficult, so the development of automated analysis methods becomes especially relevant.

Synchronization/desynchronization of periodic processes in the cell is considered one of the key mechanisms for maintaining the homeostasis of living systems under changing conditions; therefore, not only the synchronization of cells at rest, but also the synchronization of cells under stress is of interest. The most studied stress effects are heat stress (heating up to 40 °C) and changes in glucose concentration, in particular hyperglycemia [75,76,77,78,79,80]. In this work, we precisely chose these influences as tests. The aim of this study was to test the applicability and adaptation of the bispectral method for quantitative automated assessment of the synchronization parameters of Ca^2+^ and NO oscillations in electrically non-excitable cells at rest and under stress.

## 2. Materials and Methods

### 2.1. Animals

The study was conducted on male BALB/c mice weighing 21–24 г. All experiments with laboratory animals were carried out in accordance with the regulatory legal act of the Ministry of Health of the Russian Federation No. 199-n “On approval of the rules of good laboratory practice”, the international legal norms specified in the European Convention ETS No. 123 “On the protection of vertebrate animals used for experiments or in for other scientific purposes” and guidelines for working with laboratory animals from the Institute of Biochemistry of the Russian Academy of Sciences No. 57.30.12.2011. Animals received drink and food *accesso libero*.

### 2.2. Cell Isolation

Isolation of endothelial cells from the microvessels of the mouse lungs and heart was carried out by the classical method of direct magnetic separation using antibodies against CD 31 and ICAM 2 [81] with minor modifications.

For one isolation procedure, three males weighing between 20 and 22 g were used. The mice were immobilized by cervical dislocation, the skin was treated with 70% ethyl alcohol, the chest was dissected with scissors, and the heart and lungs were removed and transferred to a container with a sterile DMEM medium on ice. Under sterile conditions, organs were transferred to a Petri dish with fresh DMEM medium, and the organs were crushed using sterile scissors for 1 min to 1 mm3 cubes and placed in a 0.2% solution of collagenase type II (Abcam, Cambridge, UK) in a final volume of 25 mL. Incubated for 1 h at 37 °C. After incubation, organ pieces were pipetted for homogenization. The resulting suspension was passed through a 70 µM cell strainer and washed with 25 mL of 20% FBS in DMEM, mixed and centrifuged for 5 min at 400 g. The supernatant was discarded, and the cells were washed with PBS followed by centrifugation.

The sediment was resuspended in 3 mL of a solution of 1% BSA in PBS, and 2 μL of rabbit polyclonal antibody ab28364 was added against CD31 (PECAM-1) (Abcam, Cambridge, UK) and incubated for 30 min at room temperature on a shaker. The cells were washed twice with 1% BSA in PBS without Ca^2+^ and Mg^2+^, and 3 mL of 1% BSA and 2 mM EGTA in PBS without Ca^2+^ and Mg^2+^ were added. We added 22.5 μL of a suspension of Dynabeads (Invitrogen, Waltham, MA, USA) magnetic particles conjugated with goat anti-rabbit IgG secondary antibodies (Abcam, Cambridge, UK), previously washed in 1% BSA in PBS, and incubated for 15 min at room temperature with gentle stirring to bind CD31+ cells to the magnetic particles. We distributed the cell suspension into three 1 mL tubes and placed the tubes in a MagJet magnetic rack pack (Thermo Fisher, Waltham, MA, USA). Next, we incubated them for 2 min, removed the solution with CD31 positive cells (CD31^+^), removed the tubes from the rack, added 1 mL of a fresh solution of 1% BSA in PBS without Ca^2+^ and Mg^2+^ to each tube, resuspended, installed in a magnetic rack, and incubated for 2 min. The washing–incubation procedure was repeated 2 more times (3 times in total) to thoroughly remove CD31 negative (CD31^−^) cells. Incubation time affects the quality of the separation. The number of IgG-labelled cells (in our case CD31+ cells) isolated by the magnetic rack increases with increasing incubation time duration. After a certain point, ‘saturation’ occurs when the main population of CD31+ cells has been isolated from the suspension of all cells. Excessively long incubations can significantly increase the overall protocol time and negatively affect the viability of the isolated cell population. Therefore, it is not advisable to increase the incubation time of the cell suspension on the magnetic rack after the onset of ‘saturation’. The optimal incubation time is selected during preliminary experiments based on the times suggested by the manufacturer and visual control by the operator of the appearance of ‘precipitation’ of cells/free magnetic particles on the test tube wall adjacent to the magnet. To separate CD31+ cells from magnetic particles, each portion of the cell suspension was resuspended in 200 μL of MDEM medium preheated to 37 °C with 1% FBS, 1 mM CaCl_2_ and 5 mM MgCl_2_, pH 7.0–7.4, adding 4 µL Release Buffer (Thermo Fisher, Waltham, MA, USA) containing DNAse type I, and incubated for 15 min at room temperature with gentle stirring. After incubation, the tubes with the cell suspension were placed in a magnetic stand for 2 min, the solution was taken and transferred to new tubes pre-treated with MDEM medium c 1% FBS, 1 mM CaCl_2_ and 5 mM MgCl_2_, pH 7.0–7.4. We placed the tubes on a magnetic stand for 2 min, collected the supernatant into a new clean tube, and added fresh 1 mL of the above medium to each tube. The procedure of incubating the suspension on a magnetic stand was repeated twice, with the selection of the supernatant containing CD 31+ cells and the addition of new medium. The preserved suspension without particles was centrifuged at 350 g for 10 min. The CD 31+ cell pellet was resuspended in DMEM/F 12 supplemented culture medium by 10% FBS, 2 mM L-glutamine, 100 U/mL penicillin, 100 μg/mL streptomycin (Gibko, Waltham, MA, USA) and endothelial cells growth supplement (Sigma Aldrich, Burlington, MA, USA). The resulting suspension was transferred into culture flasks with the surface pre-treated with 0.1% gelatin (Sigma Aldrich, Burlington, MA, USA).

Cultivation was carried out according to the standard protocol in a CO_2_ incubator at 5% CO_2_ in the cultivation medium described above. Subculturing was carried out according to a standard protocol with a confluency of ~80%. Cells of 7–10 passages were used in the experiments. The purity of the cell culture was checked by staining with antibodies to CD31. Survival was monitored using propidium iodide staining. The experiments used samples with a survival rate of at least 95% [54].

### 2.3. Fluorescence Microscopy

The concentrations of Ca^2+^ and NO in cells were evaluated by staining with fluorescent probes 5 μM Fura-2 AM and 8 μM DAF-FM (Thermo Scientific, Waltham, MA, USA), respectively, for 2 h in a CO_2_ incubator in a culture medium without serum and phenol red, then washed three times with complete Hank’s solution. Cell fluorescence was measured using a fluorescent LED imaging system described earlier [54,82]. Registration of dynamic signals was carried out at wavelengths of exciting light of 340, 380 and 490 nm, switched on alternately. The illumination intensity was adjusted by setting the current values: 200 mA for 340 nm, 40 mA for 380 nm, 80 mA for 490 nm. Recording was carried out with an exposure of 0.5 s/frame and a constant gain of 323, and a 2 × 2 pixel binning was used to increase the signal-to-noise ratio. The level of cytoplasmic calcium was assessed using the ratio F340/F380 with preliminary subtraction of background values.

Cytoplasmic level NO was assessed by F490 intensity with preliminary subtraction of background values. Since the DAF-FM probe binds to NO irreversibly, NO oscillations were assessed by the rate of NO synthesis; for this purpose, the value of the derivative dNO/dt was calculated.

During the entire experiment, the cells were kept on a thermostatic stage. The recording was carried out for 15 min at a temperature of 37 °C and 20 min when heated to 40 °C, the last 15 min were analyzed in order to exclude transient processes from the analysis. At least 200 cells were analyzed in each experiment. Recording and primary processing of the obtained data were carried out using WinFluorXE v 3.8.7 8-12-16 software (J. Demster, Strathclide Electrophysiology Software, Glasgow, UK). Time series data were obtained using ImageJ v 1,54f (NIH, Bethesda, MD, USA).

### 2.4. Data Analysis

For the primary analysis of the amplitude–frequency characteristics, the canonical wavelet transform method for human and animal physiology was used. In this study, algorithms for complex transforms based on Morlet wavelet were used [83]. Next, cells with calcium and NO oscillations were selected from the entire population using the threshold algorithm described in previous work [84]. For further analysis, the bispectral analysis method was used [50]. The necessary calculations were performed in the MatLab program.

### 2.5. Bispectral Analysis Method

The basis was taken as a modification of the bispectral analysis method [50]. A detailed description of the derivation of equations and the data obtained with their help is given in the review [52] and the results in Section 3.1. Calculations and construction of bispectral maps were performed in the MatLab program using code created on the basis of BispectrumTemp published in the public domain [50]. The code modification consisted of connecting GPU calculations, implementing batch processing and saving intermediate calculation results into text files for further analysis by external auxiliary programs.

### 2.6. Statistical Processing

Experimental data were processed using nonparametric statistics methods. Results were presented as medians and percentiles of 10, 25, 75 and 90%. The statistical significance of differences between sample median values was assessed using Kruskal–Wallis analysis of variance ANOVA followed by Tukey’s test for multiple comparisons (SigmaPlot v 10) for multiple comparisons or the Mann–Wintney test for paired comparisons. Differences between sample values were considered statistically significant at a significance level of *p* < 0.05.

## 3. Results

### 3.1. Application of Bispectral Method for Non-Lineal System Analisys

The basis of bispectral analysis is the calculation of integral complex correlation characteristics (accurate to the notations) based on the formulas from [50]:

(1) $$\Phi_{1}(t,\omega_{1})=A_{1}(t,\omega_{1})e^{j\varphi_{1}},\varphi_{1}=(\omega_{1}t+\phi_{1}).$$

(2) $$\Phi_{2}(t,\omega_{2})=A_{2}(t,\omega_{2})e^{j\varphi_{2}},\varphi_{2}=(\omega_{2}t+\phi_{2}).$$

(3) $$\Phi_{3}(t,\omega_{1}+\omega_{2})=A_{3}(t,\omega_{1}+\omega_{2})e^{j\varphi_{3}},\varphi_{3}=((\omega_{1}+\omega_{2})t+\phi_{3}).$$

(4) $$\begin{array}{l} \rho_{123}(\omega_{1},\omega_{2})=\int_{-\infty }^{\infty }\Phi_{1}(t,\omega_{1})\Phi_{2}(t,\omega_{2})\bar{\Phi_{3}(t,\omega_{1}+\omega_{2})}dt=\\ =\int_{-\infty }^{\infty }A_{1}A_{2}A_{3}e^{j(\varphi_{1}+\varphi_{2}-\varphi_{3})}dt=\int_{-\infty }^{\infty }A_{1}A_{2}A_{3}e^{j\Delta \varphi }dt \end{array}$$

(5) $$\Delta \varphi =\phi_{1}+\phi_{2}-\phi_{3}-t(\omega_{1}+\omega_{2}-(\omega_{1}+\omega_{2}))=\phi_{1}+\phi_{2}-\phi_{3}.$$

where *ρ*_123_ is the bispectrum coefficient actually calculated for frequencies ω_1_ and ω_2,_ and for the three synchronously recorded signals Φ_1_, Φ_2_, Φ_3_, they are complex wavelet decomposition coefficients determined at time t and for frequencies ω_1_, ω_2_ and ω_1_ + ω_2_, respectively.

Equations (1)–(3) define the complex representation of the wavelet coefficients Φ_1_, Φ_2_, Φ_3_ in terms of moduli and complex exponents, as well as the form of phases for ease of further description. Equation (4) defines the integral form of the calculation of the bispectrum coefficient ρ_123_ as an integral over time of the product of the corresponding wavelet coefficients. Equation (4) introduces the phase shift, and expression (6) shows the automatic frequency compensation required to obtain a constant phase shift. In general, the integral in Equation (4) is a kind of complex correlation, and the integration result depends both on the simultaneous presence of significant values of *A*_1_, *A*_2_, and *A*_3_, and on the maintenance of a constant phase Δ*ϕ* over the entire integration interval.

When considering a more specific case of analyzing two signals, it is obvious that two options can be offered for calculating the bispectrum:

(6) $$\rho_{121}(\omega_{1},\omega_{2})=\int_{-\infty }^{\infty }\Phi_{1}(t,\omega_{1})\Phi_{2}(t,\omega_{2})\bar{\Phi_{1}(t,\omega_{1}+\omega_{2})}dt,$$

(7) $$\rho_{122}(\omega_{1},\omega_{2})=\int_{-\infty }^{\infty }\Phi_{1}(t,\omega_{1})\Phi_{2}(t,\omega_{2})\bar{\Phi_{2}(t,\omega_{1}+\omega_{2})}dt,$$

**Figure 1:**
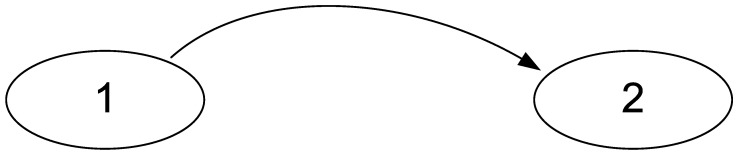
A simple scheme of signal transmission in a physiological system. Single cells can act as nodes when recording a culture of endothelial cells using microfluorometric analysis of intracellular calcium or individual brain structures when studying activity using electroencephalography.

Moreover, it is obvious that these formulas are not symmetrical with respect to the permutation of signals. This asymmetry allows us to solve the problem of determining the source and receiver of the signal. Let us consider a simple signal transmission circuit consisting of two nodes “1” and “2” (Figure 1):

Let us assume that

1. Synchronous registration of signals occurs both from node “1” and from node “2”,
2. Each node has its own generator of harmonic oscillations with characteristic frequencies ω_1_ and ω_2_, respectively,
3. There is a channel for transmitting oscillations from node “1” to node “2”, and in node “2”, the linear summation of oscillations “1” and “2” occurs,
4. Analog nonlinear signal conversion occurs at the registration stage in each node.

**Figure 2:**
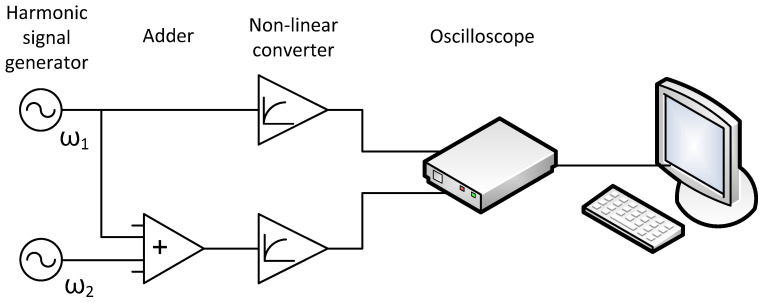
Signal transduction diagram for Figure 1.

Taking into account all the above assumptions, the simplest signal conversion circuit may look like this (Figure 2):

At the generator level, the signals look like:

(8) $$A_{1}\sin\omega_{1}t,$$

(9) $$A_{2}\sin\omega_{2}t,$$

where *A*_1_ and *A*_2_ are the amplitudes of the corresponding signals,

At the inputs of nonlinear converters, the signals look like:

(10) $$x_{1}(t)=A_{1}\sin\omega_{1}t,$$

(11) $$x_{2}(t)=kA_{1}\sin\omega_{1}t+A_{2}\sin\omega_{2}t,$$

where *k* is the signal transmission coefficient from the first node to the second. We take into account the nonlinearity of the converter in the form of a Taylor series expansion to a quadratic term of the form:

(12) $$f(x_{i})=x_{i}+Bx_{i}^{2},$$

here we have neglected the constant component of the signal, and thus, the signal recorded from the first node will have the form:

(13) $$f_{1}(t)=A_{1}\sin\omega_{1}t+B_{1}{A_{1}}^{2}\sin^{2}\omega_{1}t=A_{1}\sin\omega_{1}t+\frac{{A_{1}}^{2}B_{1}}{2}-\frac{{A_{1}}^{2}B_{1}}{2}\cos(2\omega_{1}t),$$

Thus, the second harmonic of the main signal will also appear in the signal recorded from the first node due to the nonlinearity of the transfer function. Of course, in a real system, nonlinearities are also possible, which will require taking into account terms of the Taylor series of a higher order, and accordingly, higher harmonics will appear.

The signal recorded from the second node will contain a much richer spectrum of vibrations:

(14) $$\begin{array}{l} f_{2}(t)=kA_{1}\sin\omega_{1}t+A_{2}\sin\omega_{2}t+B_{2}{\left(kA_{1}\sin\omega_{1}t+A_{2}\sin\omega_{2}t\right)}^{2}=\\ =kA_{1}\sin\omega_{1}t+A_{2}\sin\omega_{2}t+B_{2}(k^{2}A_{1}^{2}\sin^{2}\omega_{1}t+A_{2}^{2}\sin^{2}\omega_{2}t+2kA_{1}A_{2}\sin\omega_{1}t\sin\omega_{2}t)=\\ =kA_{1}\sin\omega_{1}t+A_{2}\sin\omega_{2}t+k^{2}A_{1}^{2}B_{2}\sin^{2}\omega_{1}t+A_{2}^{2}B_{2}\sin^{2}\omega_{2}t+\\ +kA_{1}A_{2}B_{2}(\cos(\omega_{1}-\omega_{2})t-\cos(\omega_{1}+\omega_{2})t), \end{array}$$

In the signal recorded from the second node, due to the nonlinearity of the transfer function, second ones will appear (harmonics of the main signals, as well as signals with a frequency equal to the sum and difference of the main frequencies.

Since the wavelet transform is essentially a frequency filtering, it is easy to determine the corresponding non-zero wavelet coefficients (excluding the coefficient for frequency **ω_1_–ω_2_** since such a frequency is usually too low to be recorded) based on Formulas (12) and (13). They are presented in Table 1:

When analyzing signal data using bispectral analysis, all the advantages of the latter appear. Further, for simplicity, we will call bispectra calculated for one signal, for example *ρ*_111_, solo-bispectra, and bispectra calculated to compare two signals, for example *ρ*_121_, cross-bispectra. When calculating the solo-bispectrum only for the first signal, we will obtain non-zero values only for frequency *ω*_1_:

(15) $$\begin{array}{l} \rho_{111}(\omega_{1},\omega_{1})=\int_{-\infty }^{\infty }\Phi_{1}(t,\omega_{1})\Phi_{1}(t,\omega_{1})\bar{\Phi_{1}(t,2\omega_{1})}dt=\int_{-\infty }^{\infty }{\left(A_{1}e^{j\omega_{1}t}\right)}^{2}\bar{\frac{1}{2}{A_{1}}^{2}B_{1}e^{j2\omega_{1}t+\pi /2}}dt=\\ =\frac{{A_{1}}^{4}B_{1}}{2}\int_{-\infty }^{\infty }e^{j(2\omega_{1}t-2\omega_{1}t-\pi /2)}dt=\frac{{A_{1}}^{4}B_{1}}{2}\int_{-\infty }^{\infty }e^{-j\pi /2}dt=\frac{{A_{1}}^{4}B_{1}}{2}e^{-j\pi /2}\int_{-\infty }^{\infty }dt. \end{array}$$

This estimate is obtained by simply multiplying the amplitudes of the corresponding harmonics of the first signal.

When calculating the solo bispectrum only for the second signal, we will obtain non-zero values for frequencies ω_1_ and ω_2_, since the signal contains their harmonics and signals with the sum of frequencies:

(16) $$\begin{array}{l} \rho_{222}(\omega_{1},\omega_{1})=\int_{-\infty }^{\infty }\Phi_{2}(t,\omega_{1})\Phi_{2}(t,\omega_{1})\bar{\Phi_{2}(t,2\omega_{1})}dt=\int_{-\infty }^{\infty }{\left(kA_{1}e^{j\omega_{1}t}\right)}^{2}\bar{\frac{1}{2}k^{2}{A_{1}}^{2}B_{1}e^{j2\omega_{1}t+\pi /2}}dt=\\ =\frac{k^{4}{A_{1}}^{4}B_{1}}{2}\int_{-\infty }^{\infty }e^{j(2\omega_{1}t-2\omega_{1}t-\pi /2)}dt=\frac{k^{4}{A_{1}}^{4}B_{1}}{2}\int_{-\infty }^{\infty }e^{-j\pi /2}dt=\frac{k^{4}{A_{1}}^{4}B_{1}}{2}e^{-j\pi /2}\int_{-\infty }^{\infty }dt,\\ \rho_{222}(\omega_{2},\omega_{2})=\int_{-\infty }^{\infty }\Phi_{2}(t,\omega_{2})\Phi_{2}(t,\omega_{2})\bar{\Phi_{2}(t,2\omega_{2})}dt=\int_{-\infty }^{\infty }{\left(A_{2}e^{j\omega_{2}t}\right)}^{2}\bar{\frac{1}{2}{A_{2}}^{2}B_{2}e^{j2\omega_{2}t+\pi /2}}dt=\\ =\frac{{A_{2}}^{4}B_{2}}{2}\int_{-\infty }^{\infty }e^{j(2\omega_{2}t-2\omega_{2}t-\pi /2)}dt=\frac{{A_{2}}^{4}B_{2}}{2}\int_{-\infty }^{\infty }e^{-j\pi /2}dt=\frac{{A_{2}}^{4}B_{2}}{2}e^{-j\pi /2}\int_{-\infty }^{\infty }dt,\\ \rho_{222}(\omega_{1},\omega_{2})=\int_{-\infty }^{\infty }\Phi_{2}(t,\omega_{1})\Phi_{2}(t,\omega_{2})\bar{\Phi_{2}(t,\omega_{1}+\omega_{2})}dt=\int_{-\infty }^{\infty }kA_{1}e^{j\omega_{1}t}{\left(A_{2}e^{j\omega_{2}t}\right)}^{2}\bar{kA_{1}A_{2}B_{2}e^{j(\omega_{1}+\omega_{2})t-\pi /2}}dt=\\ =k^{2}{A_{1}}^{2}{A_{2}}^{2}B_{2}\int_{-\infty }^{\infty }e^{j(\omega_{1}t+\omega_{2}t-\omega_{1}t-\omega_{2}t+\pi /2)}dt=k^{2}{A_{1}}^{2}{A_{2}}^{2}B_{2}\int_{-\infty }^{\infty }e^{j\pi /2}dt=k^{2}{A_{1}}^{2}{A_{2}}^{2}B_{2}e^{j\pi /2}\int_{-\infty }^{\infty }dt. \end{array}$$

When calculating the cross bispectra to determine the relationship of signals, we obtain the following non-zero cases:

(17) $$\begin{array}{l} \rho_{121}(\omega_{1},\omega_{1})=\int_{-\infty }^{\infty }\Phi_{1}(t,\omega_{1})\Phi_{2}(t,\omega_{1})\bar{\Phi_{1}(t,2\omega_{1})}dt=\int_{-\infty }^{\infty }A_{1}e^{j\omega_{1}t}kA_{1}e^{j\omega_{1}t}\bar{\frac{1}{2}{A_{1}}^{2}B_{1}e^{j2\omega_{1}t+\pi /2}}dt=\\ =\frac{k{A_{1}}^{4}B_{1}}{2}\int_{-\infty }^{\infty }e^{j(2\omega_{1}t-2\omega_{1}t-\pi /2)}dt=\frac{k{A_{1}}^{4}B_{1}}{2}\int_{-\infty }^{\infty }e^{-j\pi /2}dt=\frac{k{A_{1}}^{4}B_{1}}{2}e^{-j\pi /2}\int_{-\infty }^{\infty }dt,\\ \rho_{122}(\omega_{1},\omega_{1})=\int_{-\infty }^{\infty }\Phi_{1}(t,\omega_{1})\Phi_{2}(t,\omega_{1})\bar{\Phi_{2}(t,2\omega_{1})}dt=\int_{-\infty }^{\infty }A_{1}e^{j\omega_{1}t}kA_{1}e^{j\omega_{1}t}\bar{\frac{1}{2}k^{2}{A_{1}}^{2}B_{2}e^{j2\omega_{1}t+\pi /2}}dt=\\ =\frac{k^{3}{A_{1}}^{4}B_{2}}{2}\int_{-\infty }^{\infty }e^{j(2\omega_{2}t-2\omega_{2}t-\pi /2)}dt=\frac{k^{3}{A_{1}}^{4}B_{2}}{2}\int_{-\infty }^{\infty }e^{-j\pi /2}dt=\frac{k^{3}{A_{1}}^{4}B_{2}}{2}e^{-j\pi /2}\int_{-\infty }^{\infty }dt,\\ \rho_{122}(\omega_{1},\omega_{2})=\int_{-\infty }^{\infty }\Phi_{1}(t,\omega_{1})\Phi_{2}(t,\omega_{2})\bar{\Phi_{2}(t,\omega_{1}+\omega_{2})}dt=\int_{-\infty }^{\infty }A_{1}e^{j\omega_{1}t}A_{2}e^{j\omega_{2}t}\bar{kA_{1}A_{2}B_{2}e^{j(\omega_{1}+\omega_{2})t-\pi /2}}dt=\\ =k{A_{1}}^{2}{A_{2}}^{2}B_{2}\int_{-\infty }^{\infty }e^{j(\omega_{1}t+\omega_{2}t-\omega_{1}t-\omega_{2}t+\pi /2)}dt=k{A_{1}}^{2}{A_{2}}^{2}B_{2}\int_{-\infty }^{\infty }e^{j\pi /2}dt=k{A_{1}}^{2}{A_{2}}^{2}B_{2}e^{j\pi /2}\int_{-\infty }^{\infty }dt. \end{array}$$

We calculate the normalization of cross-bispectra by dividing into the corresponding modules of bispectra for signals:

(18) $$\begin{array}{l} \frac{\rho_{121}(\omega_{1},\omega_{1})}{\sqrt[{3}]{{\rho_{111}}^{2}(\omega_{1},\omega_{1})\rho_{222}(\omega_{1},\omega_{1})}}\approx \frac{A_{1}kA_{1}{A_{1}}^{2}B_{1}}{2\sqrt[{3}]{\frac{{A_{1}}^{8}{B_{1}}^{2}}{4}\frac{k^{4}{A_{1}}^{4}B_{2}}{2}}}=\sqrt[{3}]{\frac{B_{1}}{kB_{2}}},\\ \frac{\rho_{122}(\omega_{1},\omega_{1})}{\sqrt[{3}]{\rho_{111}(\omega_{1},\omega_{1}){\rho_{222}}^{2}(\omega_{1},\omega_{1})}}\approx \frac{A_{1}kA_{1}k^{2}{A_{1}}^{2}B_{2}}{2\sqrt[{3}]{\frac{{A_{1}}^{4}B_{1}}{2}\frac{k^{8}{A_{1}}^{8}{B_{2}}^{2}}{4}}}=\sqrt[{3}]{\frac{kB_{2}}{B_{1}}},\\ \frac{\rho_{122}(\omega_{1},\omega_{2})}{\sqrt[{3}]{\rho_{111}(\omega_{1},\omega_{1})\rho_{222}(\omega_{1},\omega_{1})\rho_{222}(\omega_{1},\omega_{2})}}\approx \frac{A_{1}A_{2}kA_{1}A_{2}B_{2}}{\sqrt[{3}]{\frac{{A_{1}}^{4}B_{1}}{2}\frac{k^{4}{A_{1}}^{4}B_{2}}{2}k^{2}A_{1}A_{2}A_{1}A_{2}B_{2}}}=\\ =\sqrt[{3}]{\frac{4{A_{2}}^{4}B_{2}}{k^{3}{A_{1}}^{4}B_{1}}}. \end{array}$$

Thus, from the ratio of peak amplitudes on the bispectrum map, one can estimate (at least in terms of “more or less”) the signal transmission coefficient *k* and the ratio of nonlinear terms *B*, i.e., an analysis of the peak values of normalized cross-bispectra makes it possible to assess the degree of nonlinearity in the analyzed system by the presence of corresponding harmonics in the signal, as well as the structure of analog processing and signal transmission in the analyzed system by the coefficient. Simply put, the presence of a peak in the bispectrum map not only indicates that the signal has corresponding frequency components, but also indicates that there is a periodic signal with a frequency equal to the sum of the frequencies of the peak position, and all these frequency components are constant relative to each other’s phase shifts. Access to the estimate of the signal transmission coefficient *k* allows one to identify conditional “receivers” and “transmitters” of signals in the analyzed system.

Depending on the hypothesis to be tested, the normalization used in the bispectral analysis can be different. We show the possibility of solving the problem of determining the signal transmission path in a network system with dedicated nodes, i.e., a cellular network of non-excitable cell culture (endotheliocytes) with a diffusion mechanism of signal transmission with Formulas (8)–(18).

### 3.2. Application of the Bispectral Analysis Method to Assess the Synchronization of Vibrations of Intracellular Signaling Molecules

**Figure 3:**
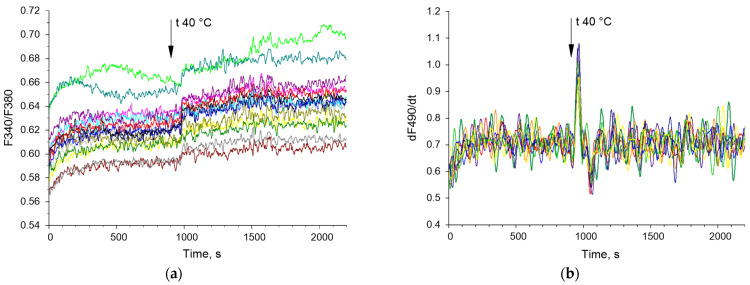
Examples of timelapses of Ca^2+^ concentration (F340/F380) (**a**) and the rate of NO synthesis (dNO/dt) (**b**) in mouse endothelial cells. Cell recordings from a randomly selected part of the field of view are shown. The vertical arrow indicates the time when the heating was turned on. Each colored curve corresponds to a dynamic series of changes in Ca^2+^ concentration (F340/F380) and NO synthesis rate (dNO/dt) in a single cell. Each colored line indicates dynamic of Ca^2+^ concentration or NO synthesis for individual cell.

**Figure 4:**
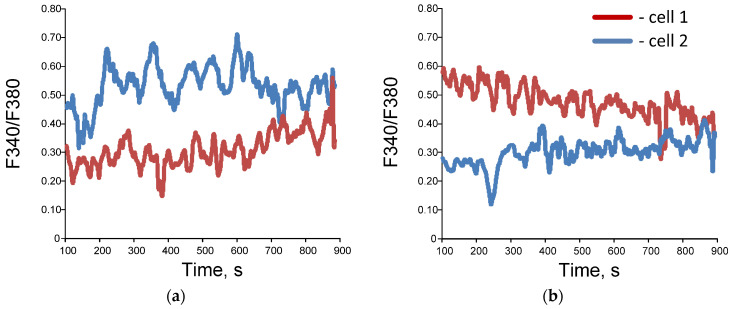

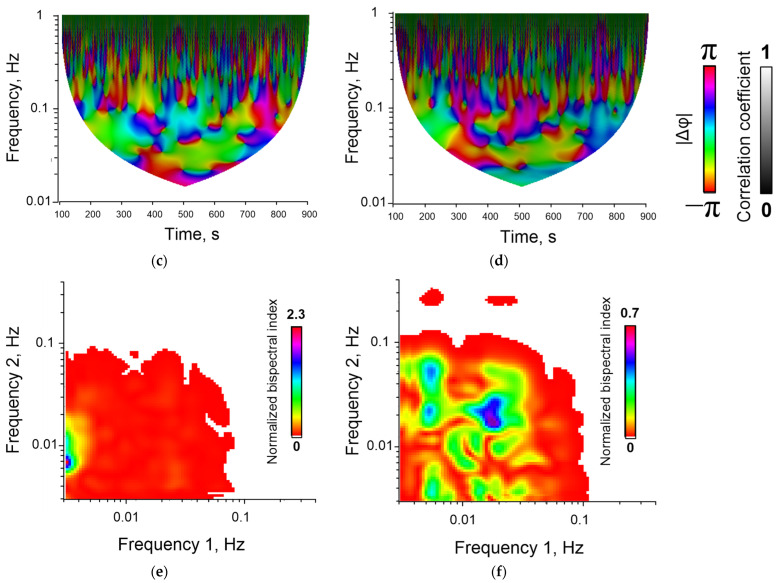
Examples of dynamic records of changes in F 340/ F 380 in single cells (**a**,**b**), wavelet coherence maps (**c**,**d**) and bispectral maps (**e**,**f**) calculated for dynamic series F340/F380 for synchronized (**a**,**c**,**e**) and unsynchronized (**b**,**d**,**f**) endothelial cells. Red and blue lines (images (**a**,**b**)) correspond to data obtained for two selected individual cells.

Examples of time series of changes in the concentration of cytoplasmic calcium and the rate of NO synthesis are shown below (Figure 3). The bispectral analysis method allows you to effectively assess the presence and degree of synchronization of oscillatory processes (Figure 4e,f) as well as the classical methods of wavelet coherence and cross-correlation (Figure 4c,d). It is noteworthy that in the case of high coherence, the bispectral analysis method reveals clearer boundaries of the frequency ranges in which synchronized oscillations occur in contrast to classical wavelet coherence (Figure 4c,e). In addition, with bispectral analysis, it is better to see whether synchronization occurs at one frequency or several.

An additional advantage of bispectral analysis is the wider dynamic range of quantitative data. The wavelet coregency and cross-correlation coefficients can take values in the range from 0 to 1. The bispectral analysis coefficient can be higher than 1 (for example, Figure 4e). This allows us to more accurately characterize the degree of synchronization across conditions and to find more subtle differences between experimental conditions.

**Figure 5:**
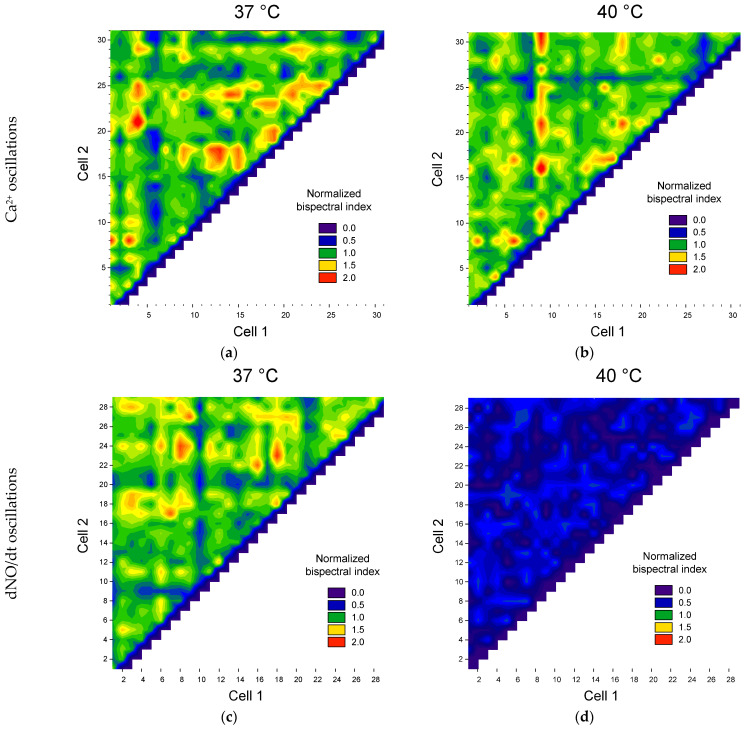
Examples of maps of the distribution of pairs of cells by the value of the maximum normalized modules of the bispectra of calcium concentration fluctuations (**a**,**b**) and the rate of NO production (**c**,**d**) at physiological temperature (**a**,**c**) and heating at 40 °C (**b**,**d**).

To quantitatively describe the degree of synchronization of cells with each other, we chose the maximum normalized bispectrum index. To test the preservation of synchronization between cells after temperature stress, we constructed matrices of bispectral coefficient values between different pairs of cells (Figure 5).

We found that temperature stress in some cells decreased the synchronization of Ca^2+^ oscillations, and in some cells, it increased (Figure 5a). At the same time, the heterogeneity of cells in synchronization with each other is observed. We observed three groups of cells. The first group is cells synchronized with a large number of other cells (red horizontal or vertical areas, Figure 5). The second group is cells with low synchronization with other cells (blue and cyan horizontal and vertical areas). The third group consists of cells moderately synchronized with each other: green and yellow areas.

Previously, we discovered the heterogeneity of cells in their ability to generate both calcium transients and oscillations of calcium and NO [54,85]. However, the heterogeneity of cells in their ability to synchronize oscillations of calcium and NO concentrations was discovered for the first time. Our results are indirectly consistent with data on significant heterogeneity of cell populations within a single culture. At the same time, cells synchronized by Ca^2+^ concentration oscillations may not be synchronized by NO oscillations and vice versa.

**Figure 6:**
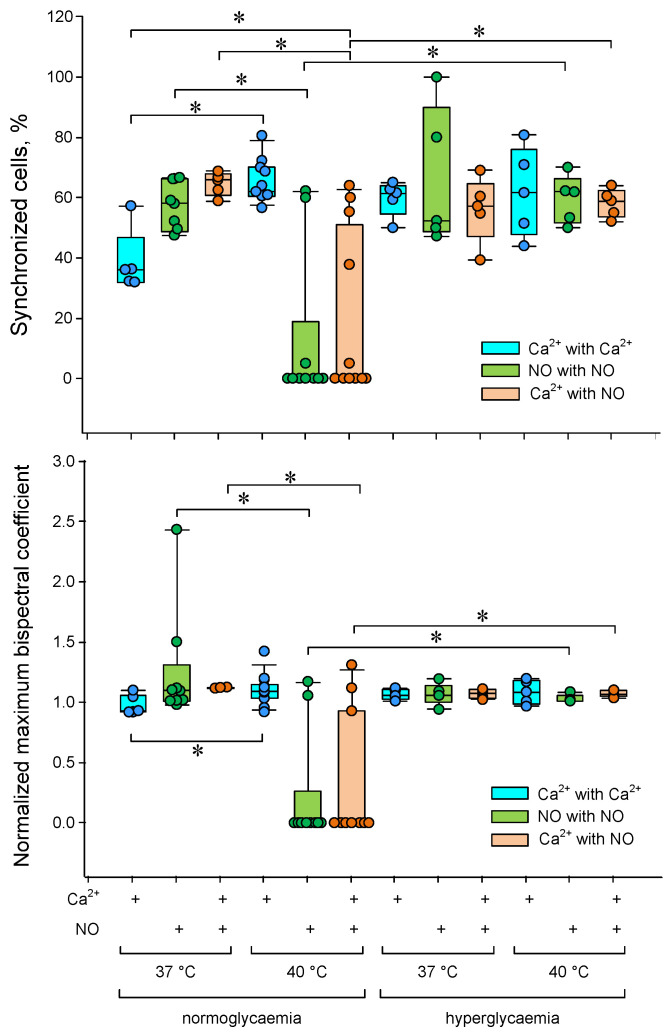
Dependence of the degree of synchronization of Ca^2+^ and NO concentrations oscillations in endothelial cells on temperature and glucose concentration. The proportions of synchronized cells are shown at the top. The average values of the normalized maximum bispectral indices for the analyzed cell population are shown below. * *p* < 0.05 Kruskal–Wallis ANOVA followed by Tukey’s test (at least 5 independent measurements).

A change in the number of cells was found with oscillations of Ca^2+^ and NO concentrations under heat stress (Figure 5b,d). To perform a quantitative assessment of the degree of cell synchronization, we used the threshold discrimination method based on the normalized maximum bispectrum index with a threshold > 1. The proportion of synchronized cells and the average value of the normalized maximum bispectral index for the entire analyzed population were chosen as quantitative characteristics of cell synchronization. We found a dependence of both the proportion of synchronized cells and the average bispectrum on the ambient temperature, glucose concentration and type of intracellular mediator (Figure 6).

The proportion of synchronized cells and the average maximum bispectrum index increased by a third with increasing temperature from 37 to 40 °C in the case of Ca^2+^ concentration oscillations. The opposite picture was observed for oscillations of the NO production rate. Additional heating sharply reduced the number of synchronized cells and the averaged maximum bispectrum. Hyperglycemia abolished the effects of the heating on the cell synchronization characteristics of calcium and nitric oxide oscillations.

Of additional interest is the assessment of the synchronization of oscillations of different mediators since this makes it possible to assess the degree of interconnection of intracellular signaling pathways with each other under different conditions.

**Figure 7:**
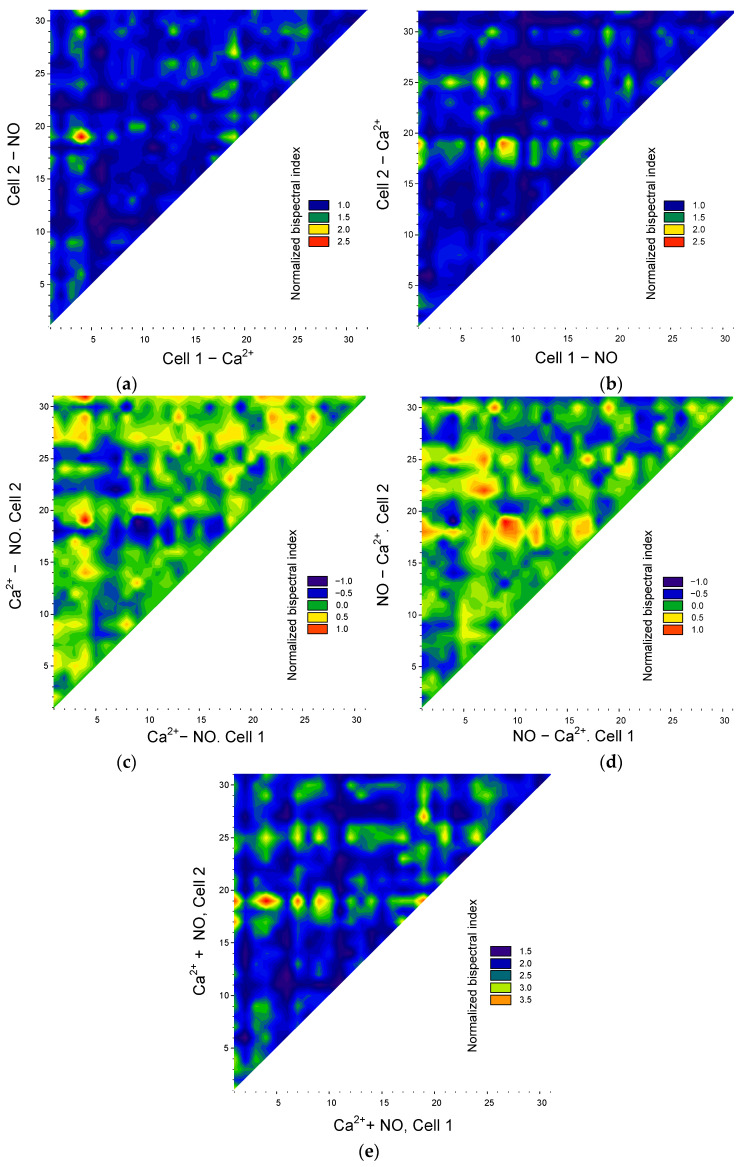
Assessing the transmission of the synchronization of Ca^2^-and NO-dependent signaling pathways in endothelial cells. Synchronization matrices of “in the direction of Ca^2+^ → NO” (**a**) or “in the direction of NO → Ca^2+^” (**b**). Matrices of differences in normalized maximum bispectral indices for oscillations of Ca^2+^ and NO (**c**) or NO and Ca^2+^ (**d**). Matrix of sums of normalized maximum bispectral indices for NO and Ca^2+^ oscillations (**e**).

In the case of “Ca^2+^-NO” synchronization, a similar picture was observed with “NO-NO” synchronization (Figure 5 and Figure 7) indicating a functional connection of signaling pathways involving calcium and NO in cell responses to hyperthermia.

It should be noted that the values of the maximum bispectral coefficient depend on the order in which the signal analysis is performed, i.e., *ρ*(*ω*_1,_*ω*_2_) ≠ *ρ*(*ω*_2,_*ω*_1_). The asymmetry of the results of bispectral analysis was mentioned above. Thus, the bispectral method makes it possible to determine the “direction” of synchronization, that is, which cell of the pair is a “transmitter” and which is a “receiver” of the synchronizing frequency. To achieve this, we constructed a series of matrices of bispectral coefficient values depending on the “direction” of analysis: from NO oscillations to Ca^2+^ oscillations or from Ca^2+^ oscillations to NO oscillations (Figure 7a,b).

From the resulting matrices, it is clear that some cells are significantly synchronized in the direction of “ Ca^2+^ → NO” (red areas at the top of Figure 7a), but at the same time, these same cells are weakly synchronized in the direction “NO → Ca^2+^” (corresponding blue areas in Figure 7b). The converse is also true. It is noteworthy that cells with pronounced synchronization in both directions of NO → Ca ^2^ and Ca^2+^ → NO were not detected at this stage of the analysis. Consequently, the cell population of endothelial cells is heterogeneous in terms of which signaling pathways are involved in their synchronization: Ca^2+^- or NO-dependent.

In addition to a general quantitative assessment, it is also possible to quantitatively analyze the relationship between potential pathways controlling the synchronization of oscillations in intracellular mediator concentrations in each specific cell. Visually, the “preponderance” between Ca^2+^ or NO synchronizations appears as a difference in the values of the maximum bispectral coefficient (Figure 7c,d). From the resulting matrices, it is clear that the cell population can be divided into three subpopulations: the first—cells with predominant synchronization of oscillations along Ca^2+^-dependent pathways (red and yellow areas, Figure 7c), the second—cells with predominant synchronization of oscillations along NO-dependent pathways (red and yellow areas, Figure 7d). Contrastingly, it is worth noting that in the first and second subpopulations, a reciprocal relationship of signaling pathways is observed. For example, between pairs with strong synchronization of Ca^2+^ oscillations, the synchronization of NO oscillations is much weaker or absent. In cells with strong synchronization of NO oscillations, Ca^2+^ synchronization is significantly weakened.

We calculated the sums of the normalized maximum bispectral coefficients to gather additional information (Figure 7e). If we assume that all cells are synchronized by one of two signaling pathways, then the sums of normalized maximum bispectral coefficients in different cells should have close values. However, we see significant cell heterogeneity three or more times in terms of the sums of bispectral coefficients. The observed phenomena can have several explanations. First, there is a subpopulation of cells with pronounced Ca^2+^- and NO-dependent synchronization, but one of them is somewhat prevalent, which is reflected in the difference matrices. Secondly, a cell can be a “receiver” in one direction, for example, “NO → Ca^2+^” and “transmitter” in another way “ Ca^2+^ → NO” (medium sum), “receivers” in both directions (low sum), and “transmitter” in both directions, but for different synchronization pairs (high sum).

**Figure 8:**
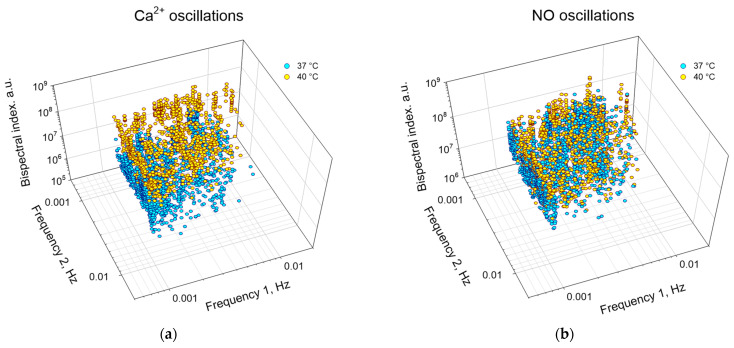
Distribution of peaks of synchronized oscillations of Ca^2+^ (**a**) or NO (**b**) by frequencies and amplitudes for the cell population of endothelial cells at a temperature of 37 °C (cyan) and 40 °C (yellow). Each point corresponds to a pair of cells to be analyzed.

In addition to assessing the degree of synchronization and the “direction of transmission” of synchronization, the bispectral analysis method can be used to quantify the frequencies and amplitudes of synchronous oscillations under different experimental conditions (Figure 8, Table 2). In particular, we found that hyperthermia increased in the first and second frequencies of synchronous oscillations of NO and Ca^2+^, respectively, compared to the control. Hyperthermia also significantly increased the frequency of synchronous oscillations, which was expressed in an increase in the absolute values of bispectral coefficients by ~20 times for Ca^2+^ oscillations and ~2 times for NO oscillations.

## 4. Discussion

The application features of the bispectral method are highly dependent on the object being studied. We propose a specific approach in the case of analyzing synchronous signals recorded from individual transmission nodes, with an attempt to answer the question: which of the nodes are the sources of oscillations, and which are the receivers. In cellular networks like cultured neurons, such a task is facilitated by the high transmission rate. In cultures of non-excitable cells, a direct answer to this question is possible using our proposed approach. We found that endothelial cells are highly heterogeneous in their ability to generate synchronized oscillations of Ca^2+^ and NO. According to mathematical models based on the Erdös–Rényi or Barabási–Albert networks, the synchronization of individual RyR receptor clusters on the endoplasmic reticulum plays an important role in the generation of calcium oscillations in the cell [86]. We hypothesize that perhaps such “auto-synchronization” for specific cells may make it a pacemaker for others. In addition, using the example of a CICR-based model, it was shown that the phenomena of multi-time scale, phase-locking, chaos and anti-phase locking transitions can be detected separately in coupled systems by varying the coupling strength [87]. Combinations of models, for example, combining a CICR-based model with a model of Ca^2+^ flow through the Na/Ca exchanger involving extracellular glutamine, also worked well in the description transmission of a “synchronizing signal” from cell to cell [70]. The participation of secreted and/or diffusible agents was suggested by us in previous work [54]. Using hippocampal neurons as an example, it has been shown that the ability to synchronize calcium oscillations depends significantly on the expression of receptors on subpopulations of cells, for example CP-AMPARs [88]. For other cell types, there may be differences in the expression of other receptors and/or the activity of intracellular signaling molecules [73,89]. It has been shown in both invertebrates and vertebrates that cell heterogeneity in the ability to generate calcium oscillations plays a key role in the development of tissues and organs of epithelial and endothelial origin [74,90]. Heterogeneity was also observed in our case, confirming the data from the literature. However, it should be noted that it is much less pronounced than in embryonic models.

Similar results were obtained in a culture of cortical neurons isolated from the cerebral cortex of rat embryos, using equal-time correlations, correlation matrix analysis and complex network analysis [91,92]. Cells have directional synchronization of Ca^2+^ oscillations, are heterogeneous in their ability to synchronize, can be combined into clusters with varying degrees of synchronization, and stress effects (mechanical stress) change the degree of synchronization and the distribution of synchronized cells [93]. However, we have not found any work assessing the synchronization of calcium and/or NO oscillations in a single population of electrically non-excitable cells. Tools based on graph theory have been widely used in the analysis of EEG data to quantitatively describe the synchronization of rhythms between cortical regions in health and disease [94]. In the future, we plan to test the possibility of using graph theory methods to improve the analysis of the cell synchronization matrices we obtain.

Data on the effects of elevated temperature on oscillations of the NO generation rate are consistent with previously obtained results and literature data [54,95,96,97,98]. However, a quantitative assessment of cell synchronization based on Ca^2+^ and NO oscillations in a population of cells has been performed for the first time in a single study. The targets of heating effects may be TRPV-, eNOS- and/or HSP-dependent signaling pathways, which ultimately regulate NO concentrations [78,79,80,99]. Unfortunately, we do not have a precise explanation for the high variation in the oscillation synchronization characteristic in the «NO with NO» group at this stage of research. We propose the following tentative explanations. First is high variation in the cell state between passages, which is encountered in biological experiments even when viability is maintained. Second, NO synthesis is regulated by fewer mechanisms (mainly PI3K/Akt- and cAMP-dependent pathways [100]) compared to calcium homeostasis (the overwhelming number of intracellular signaling pathways are Ca^2+^-dependent [101]). The smaller number of regulatory mechanisms makes NO fluctuations more sensitive to external stress effects compared to fluctuations in Ca2+ concentrations. Further studies will be required to identify and elucidate the underlying causes of the considerable heterogeneity observed in cells with respect to their NO oscillation characteristics. Differences between the directions of Ca^2+^ → NO or NO → Ca^2+^ synchronization in different cells indicate significant heterogeneity of the cell population of endothelial cells, which is consistent with literature data [54,102]. The presence of “directional synchronization” and “pacemakers” among endothelial cells is also described in the literature and is explained by differences in intracellular signaling pathways dominant in the pacemaker cell and neighboring cells [74,90,103]. The possibility of integrating Ca^2+^ and NO oscillations into cyclic signaling pathways with different participants is confirmed by literature data [53,104]. Unfortunately, at this stage, we cannot say for sure which extracellular agents are responsible for cell synchronization. Agents known from the literature are endothellin-1, acetylcholine, reactive oxygen species, NO and its forms and other signaling molecules [105,106,107,108,109,110,111,112]. Therefore, subpopulations of endothelial cells with different directions of Ca^2+^ → NO or NO → Ca^2+^ synchronization should have different “sensitivities” to different extracellular mediators and differ in their ability to secrete/release signaling molecules. At a minimum, different cells should have different levels of receptor expression for the respective agents. Experimental verification of these assumptions will be a task for future research.

We found that hyperglycemia abolishes the effects of heat testing on NO fluctuations. The effects of hyperglycemia on the characteristics of NO concentration fluctuations in cells are consistent with literature data [95]. However, we did not find any experimental studies in which the effects of hyperglycemia on cell synchronization were analyzed. It is noteworthy that in the early stages of the development of type 2 diabetes mellitus, accompanied by hyperglycemia, there is an increase in NO production by endothelial cells [113]. In our case, the abolition of the effect of the heating and the preservation of the synchronization between the cells in NO oscillations after heating could be a consequence of this phenomenon.

Recently, nanotechnologies based on nanobiotechnologies are finding more and more applications in medicine and economic activities [114,115]. However, in some cases, nanoparticles can have a cytotoxic effect through Ca^2+^-dependent signaling pathways [116]. In addition, there is increasing interest in the use of nanoparticles for the delivery of nitric oxide in agriculture and medicine [116,117,118]. Effects on cellular physiology need to be fully investigated to rule out risks of cytotoxicity. We believe that studying the effects of nanoparticles and nanomaterials on cell–cell interactions (and cell synchronization) will allow a better assessment of their potential cytotoxicity and avoidance of adverse effects.

In addition, we have shown that an increase in temperature changes the frequency response of synchronized oscillations (Table 2). We believe that this is important information that allows us to obtain additional information about the modification of intracellular signaling pathways and the set of secreted “synchronizing” molecules into the extracellular environment. Changes in the frequency and amplitude of synchronized oscillations may indicate a shortening/lengthening of the chain of signaling events in the cell and a change in the expression/activity of individual signaling components [53,55].

There are many methods and approaches to analyzing the synchronization of oscillatory processes, each of which has its own advantages and disadvantages. The classical methods of synchronization estimation are phase coherence and instantaneous phase estimation [119]. These methods allow us to quickly assess the presence of synchronization between processes, but have a number of significant limitations: they do not provide information on the amplitude of synchronized oscillations and do not allow the separation of synchronization and modulation [120]. The cross-spectral (cross-correlation) technique takes into account both amplitude and phase relationships. Estimation of instantaneous phases requires complex data preparation in advance [119]. The other common methods are the S estimator, global field synchronization, stochastic event synchrony, correlation matrix analysis and complex network analysis, etc. [41,42,43,44,45,46,47,48,91,92,121]. The S estimator allows us to accurately determine time delays of synchronized signal transmission, but it is highly sensitive to artefacts and noise, requires extremely high purity of recording or signal preprocessing. Global field synchronization is well suited for the analysis of several parallel processes, allows estimating the synchronization frequency with high accuracy, and requires low computational power [41]. The disadvantages of the method include the need to precalculate the synchronization correlation between two signals and the inability to accurately quantify the strength of the correlation. Stochastic event synchrony estimates both the frequency and amplitude of synchronized oscillations with high accuracy and has high reproducibility but requires large computational power. Correlation matrix analyses and complex network analyses allow us to estimate the strength of coupling between structures with oscillations to determine their functional connectivity and effective connectivity and to estimate the localization of sources of synchronized oscillations and their association in domains. Unlike the bispectral analysis method, the methods of correlation matrix analysis and complex network analysis do not allow us to estimate the ‘direction of signal transmission’. Synchronization between a large number of oscillators interacting in random order can be described by the Kuramoto–Daido coupling and the Winfree coupling [122]. This approach allows us to quickly and accurately describe the average phase shift and change in coupling strength in a population of oscillators. However, this approach does not allow the analysis of pairs of oscillators in the population individually. Knowledge about driver–response relationships from synchronization patterns is used to estimate the direction of transmission of a synchronized signal [123]. This approach allows us to accurately assess the contribution of noise to the synchronization of oscillatory processes, both at the ‘transmitter’ and ‘receiver’ stages, and to predict the further behavior of the system in the future. In biology, this approach is successfully implemented to analyze the number of animal populations in aquatic ecosystems [124,125]. We did not find any works on cell biology using this method. A comparison of the informativeness of learning about driver–response relationships and bispectral analysis in the study of cell populations is a task of future studies.

Despite all the advantages of the bispectral analysis method, it has several disadvantages. The first is the need for high computing power. This requirement especially grows with an increase in the number of analyzed cells according to the quadratic law. The second is a large amount of significantly varying data in the form of a three-dimensional matrix, for which it is difficult to find a single analysis algorithm. In this work, we applied an algorithm for an automated search for the maximum value of the bispectral coefficient with the determination of the X and Y coordinates of a given peak in each bispectral map. To reduce data variation, we normalized the bispectrum indicators to the average value of the indicator over the entire map.

Thus, we obtained matrices of normalized maximum values of bispectrum indexes, which allowed us to perform further analysis of the obtained data. If the peaks on the bispectral map are well localized, then we can recommend identifying regions of interest (ROI) on the bispectral maps and subsequently performing more thorough analyses (averaging, searching for median values, constructing distribution diagrams, frequency integrals, etc.) and applying methods like graph theory, etc. We plan to check the adequacy of these approaches for specific solutions to our problems in further studies.

Available computing power can be increased through performance optimization. The initial optimization of the bispectral analysis method involves performing a fast Fourier transform (FFT) at the initial stages of calculations. In view of this, calculations can be accelerated by using an optimal FFT calculation library or changing the hardware settings to solve this problem [126,127,128]. In addition, libraries that use GPU to accelerate FFT calculations have been successfully developed [129].

Practical applications of the bispectral method may include medicine and agriculture. Applications in medicine may include the search for new therapeutic approaches in the treatment of neuronal pathologies (epilepsy, Parkinson’s disease), a non-invasive assessment of blood–brain barrier permeability, and cardiovascular diseases, as well as the development of chronomodulated cancer radiotherapy strategies aimed at specific removal of cancer cells by selecting the optimal frequency and doses of radiation exposure [130,131,132,133,134]. Ca^2+^ oscillations and their synchronization in plant cells play a significant role in plant adaptation to abiotic stresses, and understanding the patterns of their control can improve stress tolerance and productivity of agricultural plants [135]. Applications to economic activities at the organismal level can include mariculture and agriculture. In particular, some of the approaches have been successfully implemented to analyze the abundance of animal populations in aquatic ecosystems, which can be used to assess their productivity and fishing potential [124,125]. The prediction of pest outbreaks can also be performed using the method of estimating the synchronization of pest population oscillations and weather conditions [136].

## 5. Conclusions

In this work, the method of bispectral analysis was adapted and successfully applied for the first time to assess the synchronization of oscillations in the concentrations of calcium and nitric oxide in the cytoplasm of electrically non-excitable cells. Using this method, we were able to quantify the proportion of synchronized cells and the strength of the synchronization. The normalized maximum bispectrum index was used to quantify the strength of cell synchronization. Heat stress (heating to 40 °C) increased the proportion of cells synchronized by oscillation of calcium concentration and decreased the proportion of cells synchronized by oscillations of NO concentration. We have shown that during hyperglycemia, the effects of heating on cell synchronization are lost. In the case of the normalized maximum bispectral index, the effects of heat stress and hyperglycemia coincided. Thus, the assessment of cell synchronization by bispectral analysis is a sensitive method for assessing the physiological state of cells. In addition to quantitative assessment, the bispectral analysis method makes it possible to evaluate the direction of synchronization transmission and to determine which cells are “generators” of the rhythm and which are receivers. We believe that the use of the bispectral analysis method will increase our knowledge of the mechanisms of generation and synchronization of biological rhythms at the cellular level, as well as find applications in personalized medicine and agriculture.

## Figures and Tables

**Table 1:** Wavelet coefficients for different frequencies for signals *f*_1_ and *f*_2_ for the circuit shown in Figure 2.

Frequency	Φ*_1_(t,ω)*	Φ*_2_(t,ω)*
ω_1_	*A_1_exp(jω_1_t)*	*kA_1_exp(jω_1_t)*
2ω_1_	*A_1_^2^B_1_exp(j(2ω_1_t + π/2))/2*	*k^2^A_1_^2^B_2_exp(j(2ω_1_t + π/2))/2*
ω_2_	*0*	*A_2_exp(jω_2_t)*
2ω_2_	*0*	*A_2_^2^B_2_exp(j(2ω_2_t + π/2))/2*
ω_1_ + ω_2_	*0*	*kA_1_ A_2_B_2_exp(j((ω_1_ + ω_2_)t − π/2))*

**Table 2:** Amplitude-frequency characteristics of synchronized oscillations of Ca^2+^ and NO.

No.	Intracellular Messenger	Ca^2+^ Oscillations		NO Oscillations	
	Temperature, °C Characteristics	37	40	37	40
1	Frequency 1, Hz × 10^−3^	2.2 (1.2; 4.2)	2.4 (1.1; 3.9)	1.9 (1.1; 3.9)	3.0 (1.6; 4.6) *
2	Frequency 2, Hz × 10^−3^	1.7 (1.2; 3.6)	2.8 (1.4; 3.9) *	2.5 (1.3; 4.2)	2.5 (1.6; 4.6)
3	Peak Value, a. u. × 10^6^	8 (5; 13)	116 (69; 170) *	51 (29; 84)	93 (60; 146) *

* *p* < 0.05 vs. 37 °C, Mann–Whitney test (at least 400 pairs of cells were analyzed for each option). Data are presented as medians with 25 and 75 percentiles. Frequency 1 or 2 corresponds to synchronization in direction cell1 → cell2 or cell2 → cell1, respectively.

## Data Availability

The raw data supporting the conclusions of this article will be made available by the authors without undue reservation.
